# Supplementary material for: Tasks and Experiences of the Prospective, Longitudinal, Multicenter MoMar (Molecular Markers) Study for the Early Detection of Mesothelioma in Individuals Formerly Exposed to Asbestos Using Liquid Biopsies
Source: Cancers (Basel). 2023 Dec 18;15(24):5896. doi: 10.3390/cancers15245896 (PMC10742125; doi:10.3390/cancers15245896)
Supplement: Supplementary file 1 [file cancers-15-05896-s001.zip › Weber 2023 Supplementary Material S1 (Cancers).pdf]

Supplementary Material S1

Determination of calretinin

Concentrations of calretinin in plasma were determined using the Calretinin ELISA kit by DLD Diagnostika GmbH (Hamburg, Germany) according to the manufacturer's instructions. All reagents and samples were equilibrated to 22 °C and the incubations were performed at 22 °C. Plasma samples (30 µl) were diluted 1:5 in the dilution buffer. All samples were determined in duplicate. Optical densities were measured using a SpectraMax 384 plus plate reader (Molecular Devices, Sunnyvale, CA, USA). The standard curve was obtained by four-parameter curve fitting using SoftMax Pro 4.7.1 (Molecular Devices).

Determination of mesothelin

Concentrations of mesothelin in plasma samples were determined using the ELISA kit MESOMARK by Fujirebio Diagnostics, Inc. (Malvern, PA, USA) according to the manufacturer's instructions with modifications as described before (39).

Statistical analyses

Calretinin and mesothelin concentrations were presented as box plots with median and interquartile range (IQR), overlaid with dot plots. Whiskers represent minimum and maximum. The two-sample Wilcoxon rank-sum test was applied to compare the distribution of calretinin and mesothelin values between prediagnostic mesothelioma cases and controls.

Biomarker classification performance was determined by receiver operating curve (ROC) with the area under curve (AUC) estimated to assess a marker's sensitivity for varying values of specificity. 95% Wald confidence intervals (CI) were calculated for the AUCs. For the

sequential combination of both markers, first calretinin was used for classification. Afterwards, mesothelin was examined for calretinin-negative subjects only. This procedure was repeated for all possible cut-points to calculate the related sensitivities and specificities resulting in a point cloud instead of a traditional ROC curve. Therefore, the AUC for this combination was calculated as descriptive measure for the “best case” scenario, so that the points with highest specificities and sensitivities were considered. The related intervals show minimum and maximum obtainable AUCs.

Statistical analyses were performed using SAS/STAT and SAS/IML software, version 9.4 (SAS Institute Inc., Cary, NC, USA) and GraphPad Prism version 9.5.1 (GraphPad Software, La Jolla California, USA) was used for generating graphs.
